# PARK7 Diminishes Oxidative Stress-Induced Mucosal Damage in Celiac Disease

**DOI:** 10.1155/2020/4787202

**Published:** 2020-09-05

**Authors:** Apor Veres-Székely, Mária Bernáth, Domonkos Pap, Réka Rokonay, Beáta Szebeni, István M. Takács, Rita Lippai, Áron Cseh, Attila J. Szabó, Ádám Vannay

**Affiliations:** ^1^1st Department of Paediatrics, Semmelweis University, 1085 Budapest, Hungary; ^2^MTA-SE Pediatrics and Nephrology Research Group, 1085 Budapest, Hungary

## Abstract

Coeliac disease (CD) is a chronic, immune-mediated small intestinal enteropathy, accompanied with gluten-triggered oxidative damage of duodenal mucosa. Previously, our research group reported an increased mucosal level of the antioxidant protein Parkinson's disease 7 (PARK7) in children with CD. In the present study, we investigated the role of increased PARK7 level on the epithelial cell and mucosal integrity of the small intestine. The presence of PARK7 was investigated using immunofluorescent staining on duodenal mucosa of children with CD and on FHs74Int duodenal epithelial cells. To investigate the role of oxidative stress, FHs74Int cells were treated with H_2_O_2_ in the absence or presence of Comp23, a PARK7-binding compound. Intracellular accumulation of reactive oxygen species (ROS) was determined by DCFDA-based assay. Cell viability was measured by MTT, LDH, and Annexin V apoptosis assays. Disruption of cytoskeleton and cell adhesion was investigated by immunofluorescence staining and by real-time RT PCR. Effect of PARK7 on mucosal permeability was investigated *ex vivo* using intestinal sacs derived from control and Comp-23-pretreated mice. Comp23 treatment reduced the H_2_O_2_-induced intracellular accumulation of ROS, thus preserving the integrity of the cytoskeleton and also the viability of the FHs74Int cells. Accordingly, Comp23 treatment increased the expression of antioxidants (*NRF2*, *TRX1*, *GCLC*, *HMOX1*, *NQO1*), cell-cycle regulators (*TP53*, *CDKN1A*, *PCNA*, *BCL2*, *BAX*), and cell adhesion molecules (*ZO1*, *CDH1*, *VCL*, *ITGB5*) of H_2_O_2_-treated cells. Pretreatment with Comp23 considerably decreased the small intestinal permeability. In this study, we demonstrate that PARK7-binding Comp23 reduces the oxidative damage of duodenal epithelial cells, via increased expression of NRF2- and P53-regulated genes. Our results suggest that PARK7 plays a significant role in the maintenance of mucosal integrity in CD.

## 1. Introduction

Coeliac disease (CD) is a chronic, immune-mediated small intestinal enteropathy triggered by the ingestion of gluten in the genetically predisposed individuals [1]. Degraded gluten peptides and the resulting chronic inflammation induce excessive accumulation of reactive oxygen species (ROS) [2], leading to the injury of the epithelial layer and the subsequent deterioration of mucosal integrity [3, 4].

Parkinson's disease 7 (PARK7) is a multifunctional molecule, which is primarily investigated in connection with neurodegenerative diseases [5, 6]; however, its possible role has been suggested in other diseases, as well [7]. Previously, our research group reported elevated expression of PARK7 in the duodenal mucosa of therapy-naive children with CD [8], and genome-wide association studies identified PARK7 polymorphisms as a predisposing factor in CD [9] and ulcerative colitis [10].

The cytoprotective effect of PARK7 can be exerted through various mechanisms. Among others, PARK7 is suggested to exert antioxidative defence directly via its enzymatic and chaperon activity; however, PARK7 influences the degree of oxidative damage more likely through its transcriptional regulatory effect. PARK7 as an oxidative sensor stabilizes transcription factors, including NRF2 and P53 in an oxidation-dependent manner. Indeed, in response to oxidative stress due to its conformation changes, PARK7 releases these transcription factors, allowing them to translocate into the nucleus and to induce the expression of stress-response elements [11, 12]. These mechanisms altogether moderate the oxidative damage of intracellular macromolecules, promote repair processes, and enhance the viability of the affected cells.

In the present study, we aimed to investigate the possible role of PARK7 in the pathomechanism of CD, with particular attention on oxidative damage of intestinal epithelial cells.

## 2. Methods

### 2.1. Duodenal Biopsies

Mucosal biopsies of pediatric therapy-naive CD patients and controls were collected at the 1st Department of Paediatrics, Semmelweis University, Hungary. Written informed consent was obtained from the parents of each participant prior to the procedure, and the study was approved by the Semmelweis University Regional and Institutional Committee of Science and Research Ethics (TUKEB 58/2013). CD was diagnosed based on the criteria of the European Society for Pediatric Gastroenterology, Hepatology, and Nutrition [13]. Controls were referred with chronic abdominal pain, growth retardation, and diarrhoea, and an upper gastrointestinal endoscopy was part of their diagnostic procedure. Biopsy samples were immediately snap-frozen and stored at −80°C until further analysis.

### 2.2. *Ex Vivo* Mucosal Permeability Measurement

All experiments were approved by the institutional committee on animal welfare (PEI/OO1/83-4/2013). Intestinal permeability was measured based on Mateer et al. and Lange et al. [14, 15]. Briefly, 2 cm long intestinal sacs were prepared from the small intestine of untreated (controls) and Comp23 ([N-[4-(8-methyl(4-hydroimidazo[1,2-a]pyridin-2-yl)) phenyl] (3,4,5-trimethoxyphenyl) carboxamide]; AKSci, Union City, CA, USA) pretreated (10 mg/kg, intraperitoneally, 1 hour before experiment) C57Bl/6J mice (*n* = 7‐9/group). The intestinal sacs were equally filled with 0.1% Evans blue diluted either in Dulbecco's modified Eagle medium (DMEM; Thermo Fisher Scientific, Waltham, MA, USA) or in DMEM supplemented with H_2_O_2_ (1000 *μ*M) and placed into 5 ml phosphate-buffered saline (PBS) at 37°C. The permeation of Evans blue was measured in every 20 minutes at 590 nm in a Hidex Chameleon Microplate Reader (Triathler, Plate Chameleon, 300SL Lablogic Systems, Inc., Brandon, FL, USA) using the MikroWin 2000 program.

### 2.3. FHs74Int Small Intestinal Epithelial Cell Culture

Human small intestinal epithelial cell line (FHs74Int (CCL-241), American Type Culture Collection, Manassas, VA, USA) was cultured in Hybri Care Medium (American Type Culture Collection) supplemented with 30 ng/ml EGF, 10% heat-inactivated fetal bovine serum (FBS) (Invitrogen, Carlsbad, CA, USA), and 1% penicillin and streptomycin mixture (Sigma-Aldrich Co., St. Louis, MO, USA) under standard cell culture conditions (37°C, humidified, 5% CO_2_/95% air environment).

For ROS assays, cells were seeded into 96-well plates at a density of 10^4^ cells/well (*n* = 5 well/treatment group) and treated with Comp23 (0.001 *μ*M) overnight, thereafter with H_2_O_2_ (50, 100, 250, 500, 1000 *μ*M) (Sigma-Aldrich) for 30 minutes. For MTT and LDH assays, cells were seeded into 96-well plates at a density of 10^4^ cells/well (*n* = 5 well/treatment group) and treated with Comp23 (0.0001, 0.001, 0.01, 0.1, 1 *μ*M) overnight, thereafter with H_2_O_2_ (1000 *μ*M) for 24 h. For Annexin V apoptosis assay, cells were seeded into 6-well plates at a density of 3 × 10^5^ cells/well and treated with Comp23 (0.001 *μ*M) overnight, thereafter with H_2_O_2_ (1000 *μ*M) for 24 hours, then measured in triplicates (*n* = 9/treatment group). For real-time RT-PCRs, cells were seeded into 96 well plates at a density of 10^4^ cells/well (*n* = 5 well/treatment group) and treated with Comp23 (0.001 *μ*M) overnight, thereafter with H_2_O_2_ (1000 *μ*M) for 24 hours. For immunocytochemistry, cells were seeded into 4 well chambers at a density of 8 × 10^4^ cells/well and treated with Comp23 (0.001 *μ*M) overnight, thereafter with H_2_O_2_ (1000 *μ*M) for 4 hours. Vehicle-treated cells served as controls in all experiments.

### 2.4. MTT Cell Proliferation Assay

MTT cell proliferation assay was performed using the Cell Proliferation Kit I (MTT) (Roche Diagnostics) according to the manufacturer's recommendations. Absorbance was recorded at 570 nm and 690 nm as background using a Hidex Chameleon Microplate Reader using the MikroWin 2000 program.

### 2.5. LDH Cytotoxicity Assay

Reagents for *in vitro* LDH cytotoxicity assay [16] were purchased from Sigma-Aldrich. Absorbance was recorded at 570 nm and 690 nm as background in a Hidex Chameleon Microplate Reader using the MikroWin 2000 program.

### 2.6. Apoptosis Detection Assay

Apoptosis assay was performed using the FITC Annexin V Apoptosis Detection Kit I (BD Pharmingen) according to the manufacturer's recommendations. Cells that are negative for Annexin V and PI were referred to as viable, cells that are positive for Annexin V and negative for PI were referred as early apoptotic, and cells that are positive for both Annexin V and PI were referred as late apoptotic cells. Flow cytometry analysis was performed using a FACS Aria cytometer (BD).

### 2.7. ROS Assay

Intracellular ROS accumulation was measured using 2′,7′-dichlorofluorescein diacetate (DCFDA, Sigma-Aldrich) fluorescent dye [17]. Cells were washed twice with PBS; thereafter, 50 *μ*l of DCFDA solution (5 *μ*M in PBS) was added for each well and incubated for 30 minutes in cell culture incubator. The fluorescence signal was measured for 30 minutes in every 5 minutes after induction of oxidative stress in the Hidex Chameleon Microplate Reader (*λ*_exc_: 485 nm, *λ*_em_: 535 nm) using MikroWin 2000 program.

### 2.8. RNA Isolation, Reverse Transcription, and Real-Time RT-PCR

Total RNA was isolated from FHs74Int cells by Geneaid Total RNA Mini Kit (Geneaid Biotech Ltd., New Taipei City, Taiwan). Equal RNA was reverse-transcribed using Maxima First Strand cDNA Synthesis Kit for RT-qPCR (Life Technologies) to generate the first-stranded cDNA. The mRNA expressions were determined by real-time RT-PCR using the LightCycler 480 SYBR Green I Master enzyme mix on a Light Cycler 480 system (Roche Diagnostics, Mannheim, Germany). The nucleotide sequences of the primer pairs are shown in Table 1. The results were analyzed by the LightCycler 480 software version 1.5.0.39 (Roche Diagnostics). The relative mRNA expression was determined in comparison with *RN18S* as an internal control using the ∆∆Ct method [18]. Data were normalized and presented as the ratio of their control values.

### 2.9. Immunofluorescence Staining

The localization of PARK7, ZO-1, and the cytoskeletal actin architecture was investigated by immunofluorescence staining on frozen biopsy samples and FHs74Int cells. After repeated washing with PBS, slides were permeabilized with Cytofix/Cytoperm (BD Pharmingen, San Diego, CA, USA) for 15 minutes at RT, washed with Perm/Wash Buffer solution (BD Pharmingen), and incubated with a primary antibody specific for PARK7 (ab18257; rabbit, 1 : 1000, Abcam, Cambridge, US), ZO-1 (ab96587; rabbit, 1 : 1000, Abcam), or Alexa Fluor® 546 phalloidin (7.5 units/mL, A22283; Thermo Fisher Scientific) for 1 hour at RT. In case of PARK7 and ZO-1 staining, slides were incubated with antirabbit Alexa Fluor 568®-conjugated secondary antibody (1 : 1000, A11036; Thermo Fisher Scientific) or antirabbit Alexa Fluor 488®-conjugated secondary antibody (1 : 1000, A21206; Thermo Fisher Scientific) for 30 minutes at RT. Thereafter, the slides were washed with a Perm/Wash Buffer solution and coverslipped with ProLong™ Gold Antifade Mountant with DAPI (Thermo Fisher Scientific). Sections were analyzed with an Olympus IX81 fluorescent microscope system.

### 2.10. Graphical Analysis of Damaged Cell Ratio

The ratio of phalloidin-stained FHs74Int cells with different cytoskeletal status was analyzed. The images were taken with 10x objective, and each cell from the field of view (*n* = 400‐500/treatment group) was categorised manually into three groups. Thereafter, cells with a healthy cytoskeleton, cells with a damaged cytoskeleton, and burst cells were counted using the ImageJ 1.48 software (The National Institutes of Health, Bethesda).

### 2.11. Statistical Analysis

The statistical evaluation of data was performed by the GraphPad Prism 6.01 software (GraphPad Software Inc., La Jolla, CA, USA). After testing normality with the Kolmogorov-Smirnov test, the raw data of real-time RT-PCR measurements were analyzed with the Mann–Whitney *U* test to determine the differences between the corresponding groups. Multiple comparisons of raw data derived from MTT, LDH, Annexin V apoptosis, ROS assays, and *ex vivo* mucosal permeability measurements were performed using multiple *t*-test and ordinary two-way ANOVA with Dunnett correction. The ratio of cells with healthy or damaged cytoskeleton after various treatments was compared using the chi-square test. *p* ≤ 0.05 was considered as statistically significant.

## 3. Results

### 3.1. The Presence of PARK7 in Duodenal Epithelial Cells

**Figure 1:**
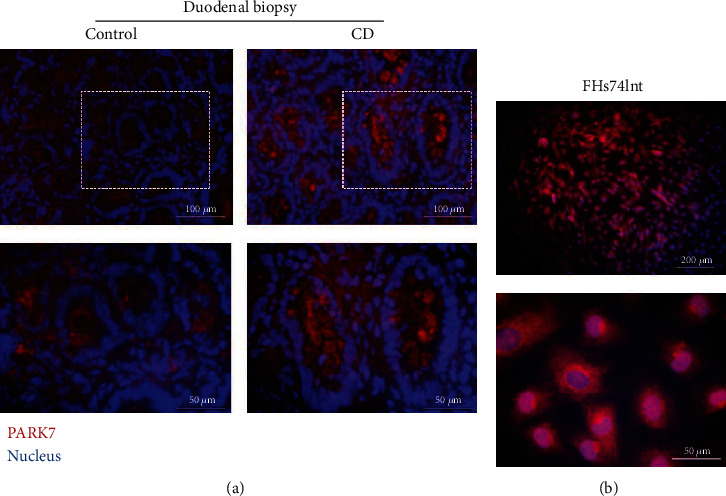
The presence of PARK7 in duodenal epithelial cells. Localization of PARK (red) was investigated by immunofluorescence staining in duodenal mucosa of children with celiac disease (CD) and controls (a) and in FHs74Int cells (b). Cell nuclei were counterstained with DAPI (blue). Scale bar: 200, 100, or 50 *μ*m.

An increased PARK7 immunopositivity was observed in the epithelial cells of the duodenal crypt and in the lamina propria of duodenal biopsies derived from children with CD compared to controls (Figure 1(a)). A definite PARK7 immunopositivity was also present in the cytoplasm and nucleus of FHs74Int cells (Figure 1(b)).

### 3.2. Comp23 Prevented Intracellular ROS Accumulation

**Figure 2:**
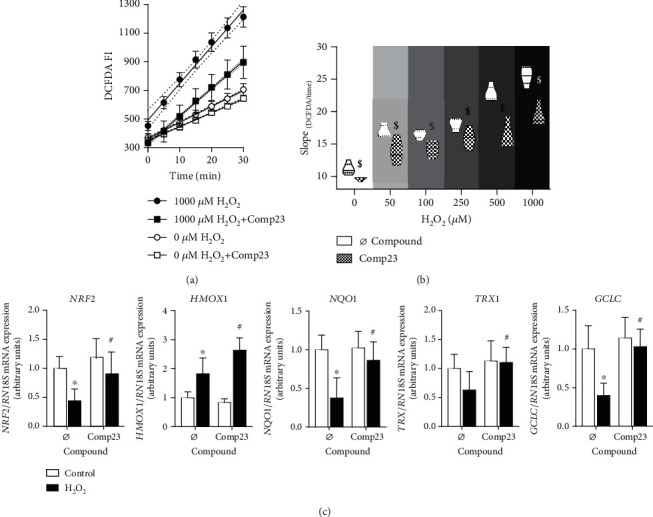
Effect of Comp23 on ROS accumulation inFHs71Int cells. FHs74Int duodenal epithelial cells were treated with different concentrations of H_2_O_2_ in the absence or presence of Comp23 (0.001 *μ*M). Scatter plot (a) serves as a representative figure of the time-related fluorescence intensity after treatment with 1000 *μ*M H_2_O_2_ with or without Comp23. Violin plot (b) indicates the slope distribution of time-related DCFDA fluorescence intensity change in the given group at different concentrations of H_2_O_2_. Relative mRNA expressions (c) at 1000 *μ*M H_2_O_2_ concentration were determined by comparison with *18S* ribosomal RNA as an internal control. Results are presented as mean + SD (*n* = 5). $*p* < 0.05 vs. Ø compound at the concerning H_2_O_2_ concentration (multiple *t*-test); ∗*p* < 0.05 vs. control+Ø compound (Mann-Whitney *U* test); #*p* < 0.05 vs. H_2_O_2_+Ø compound (Mann–Whitney *U* test).

The effect of Comp23 on the H_2_O_2_-induced ROS accumulation in the FHs74Int duodenal epithelial cells was investigated by DCFDA-based ROS assay (Figures 2(a) and 2(b)). The treatment with H_2_O_2_ resulted in an increasing accumulation of ROS in a dose-dependent manner, which was reduced by 0.001 *μ*M Comp23 at each H_2_O_2_ concentration.

The oxidative stress induced by H_2_O_2_ resulted in decreased *NRF2*, *NQO1*, and *GCLC* and elevated *HMOX1* mRNA levels, while cotreatment with Comp23 increased the expression of *NRF2*, *HMOX1*, *NQO1*, *TRX1*, and *GCLC* (Figure 2(c)).

### 3.3. Comp23 Protected Cells against Oxidative Stress-Induced Cytotoxicity

**Figure 3:**
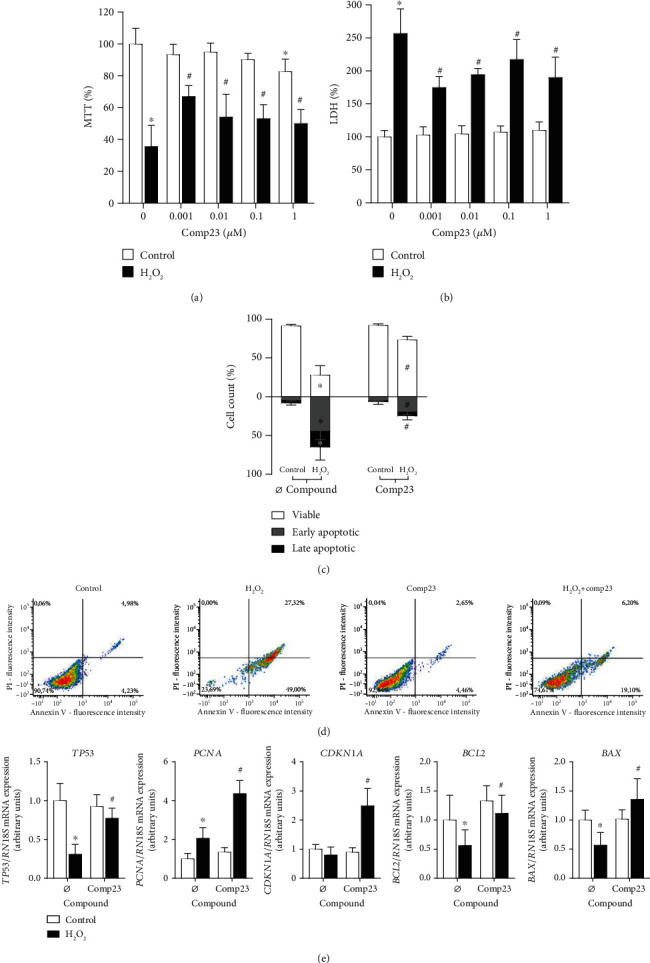
Effect of Comp23 on the H_2_O_2_-induced cell death of FHs71Int cells. FHs74Int duodenal epithelial cells were treated with different concentrations of Comp23 in the absence or presence of H_2_O_2_ (1000 *μ*M); then, oxidative stress-induced cytotoxicity was determined by MTT (a), LDH (b), and Annexin V apoptosis ((c, d); 0.001 *μ*M Comp23) assays. Relative mRNA expressions (e) at 0.001 *μ*M Comp23 concentration were determined by comparison with *18S* ribosomal RNA as an internal control. Results are presented as mean + SD ((a, b, e): *n* = 5; (c): *n* = 9). ^∗^*p* < 0.05 vs. control+Ø compound ((a, b, c): multiple *t*-test; (e): Mann-Whitney *U* test); #*p* < 0.05 vs. H_2_O_2_+Ø compound ((a, b, c): two-way ANOVA; (e): Mann-Whitney *U* test).

The effect of Comp23 treatment on the H_2_O_2_-induced cytotoxicity of FHs74Int duodenal epithelial cells was investigated by MTT, LDH, and Annexin V apoptosis assays. The treatment with H_2_O_2_ resulted in decreased viability, which was significantly restored by Comp23 at a wide concentration range (Figure 3(a)). Accordingly, the enzyme activity of LDH, released from the injured cells, was significantly lower in the supernatant of Comp23-treated cells (Figure 3(b)). Similarly, the Comp23 treatment significantly reduced the percentage of apoptotic cells in the H_2_O_2_-treated group (Figures 3(c) and 3(d)).

The oxidative stress induced by H_2_O_2_ resulted in decreased *TP53*, *BCL2*, and *BAX* and elevated *PCNA* mRNA levels, while cotreatment with Comp23 increased the expression of *TP53*, *PCNA*, *CDKN1A*, *BCL2*, and *BAX* (Figure 3(e)).

### 3.4. Comp23 Improved the Cytoskeletal Status under Oxidative Conditions

**Figure 4:**
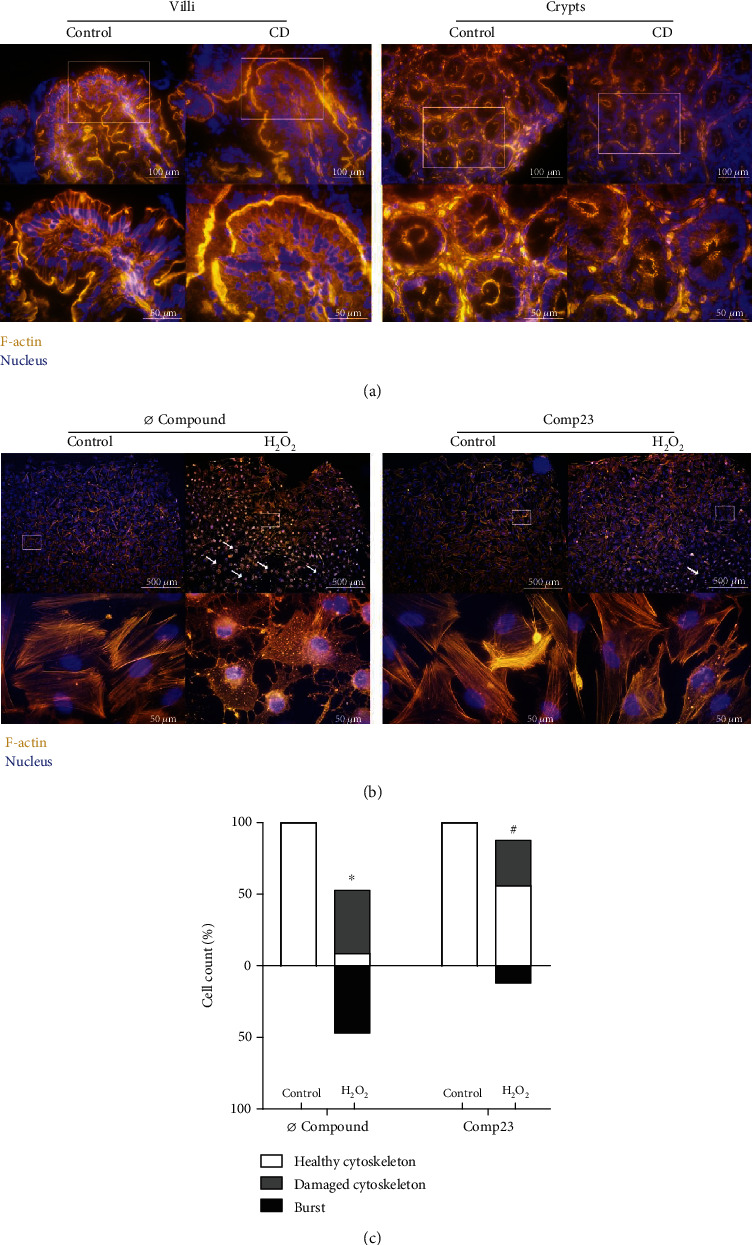
Cytoskeletal disruption of intestinal mucosa in celiac disease (CD) and of duodenal epithelial cells. Actin filament structure was investigated by phalloidin (orange) staining in duodenal mucosa of children with CD and controls (a) and in FHs74Int duodenal epithelial cells (b) after treatment with H_2_O_2_ (1000 *μ*M) in the absence or presence of Comp23 (0.001 *μ*M). Cell nuclei were counterstained with DAPI (blue). White arrows indicate burst cells. Scale bar: 500 *μ*m, 100 *μ*m, and 50 *μ*m. The percent of intact and damaged cells (c) was determined by graphical analysis. Results are presented as the percentage of total cells in a field of view (*n* = 400‐500/treatment group). ^∗^*p* < 0.05 vs. control+Ø compound; #*p* < 0.05 vs. H_2_O_2_+Ø compound (chi-square test).

The architecture of the actin cytoskeleton of mucosal epithelial cells was visualized by phalloidin staining in duodenal biopsy samples of children with CD and controls and also in FHs74Int epithelial cells (Figure 4(a)). The fluorescence signal was intense and continuous at the apical, basal, and lateral surface of the epithelial cells both in the villi and in the crypts of the control children. However, the healthy architecture of the actin cytoskeleton was disturbed in the mucosal epithelial cells of children with CD, and instead, granular staining appeared in the cytoplasm. The H_2_O_2_ treatment disrupted the actin filaments of the FHs74Int cells shrinking them into intracellular dots. In some cells, oxidative stress led to cell death as indicated by the increased number of burst cells (white arrows). Comp23 treatment of the cells largely reduced the signs of oxidative damages, the structure of the actin cytoskeleton remained mainly intact, and only a few granular dots were present (Figure 4(b)). The ratio of damaged and dead cells was also reduced by Comp23 after treatment with H_2_O_2_ (Figure 4(c)).

### 3.5. Comp23 Prevented Cell Adhesion Damage Induced by Oxidative Stress

**Figure 5:**
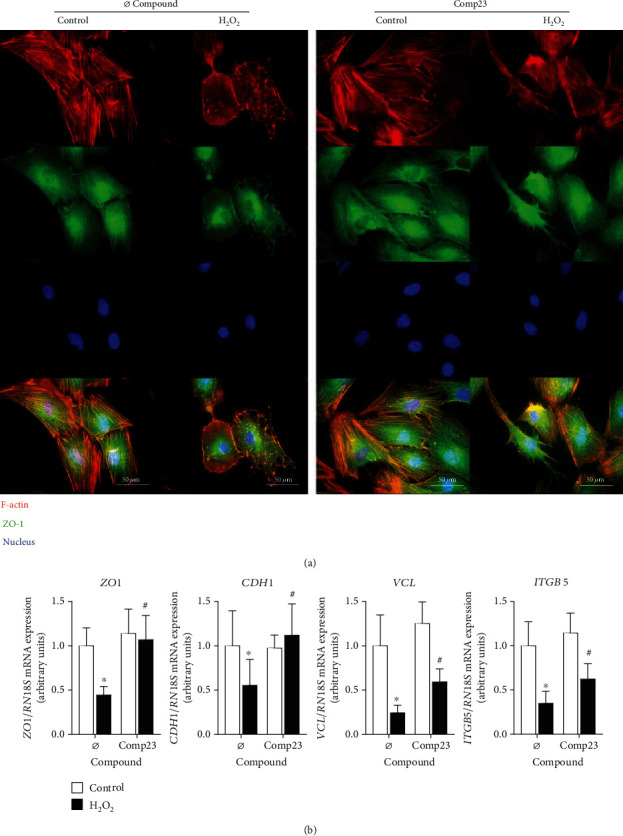
Damage of cell adhesion in epithelial cells induced by oxidative stress. Colocalization of actin (red) and zonula occludens 1 (ZO-1, green) was investigated by immunofluorescence staining (a) in FHs74Int duodenal epithelial cells after treatment with H_2_O_2_ (1000 *μ*M) in the absence or presence of Comp23 (0.001 *μ*M). Cell nuclei were counterstained with DAPI (blue). Scale bar: 50 *μ*m. Relative mRNA expressions (b) in FHs74Int cells were determined by comparison with *18S* ribosomal RNA as an internal control. Results are presented as mean + SD (*n* = 5). ^∗^*p* < 0.05 vs. control+Ø compound (Mann-Whitney *U* test); #*p* < 0.05 vs. H_2_O_2_+Ø compound (Mann-Whitney *U* test).

Localisation of actin cytoskeleton and cell adhesion molecules was visualized by phalloidin and immunofluorescence staining of the FHs74Int cells, respectively (Figure 5(a)). In control cells with healthy cytoskeleton, ZO-1 co-localized with the actin filaments. In H_2_O_2_ treated cells actin fibers aggregated into dots and ZO-1 was released from cytoskeleton and dispersed into cytoplasm. These effects of oxidative stress were diminished by the Comp23-treatment of the cells. Similarly, Comp23 treatment increased the expression of *ZO1*, *CDH1*, *VCL* and *ITGB5* cell adhesion molecules (CAMs) of H_2_O_2_ treated FHs74Int cells (Figure 5(b)).

### 3.6. Comp23 Prevented the Mucosal Permeability

**Figure 6:**
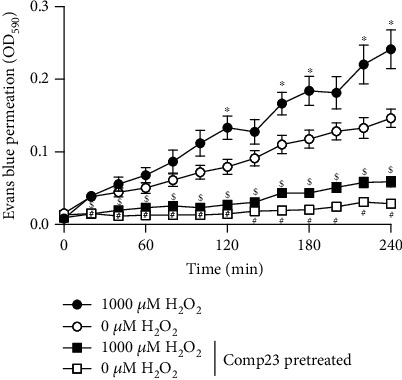
Mucosal permeability of small intestinal sacs of untreated and Comp23-treated mice. Permeability of small intestinal sacs derived from control and Comp23 pretreated mice, filled with 0.1% Evans blue diluted in DMEM were investigated in the absence or presence of H_2_O_2_ (1000 *μ*M). The permeation of Evans blue was measured in every 20 minutes at 590 nm. The results are presented as mean ± SEM (*n* = 7‐9). ^∗^*p* < 0.05 0 *μ*M H_2_O_2_ vs. 1000 *μ*M H_2_O_2_; #*p* < 0.05 0 *μ*M H_2_O_2_ vs. 0 *μ*M H_2_O_2_+Comp23; $*p* < 0.05 1000 *μ*M H_2_O_2_ vs. 1000 *μ*M H_2_O_2_+Comp23 at the concerning time (two-way ANOVA).

Intestinal sacs prepared from the small intestine of the control and Comp23-pretreated mice were used to investigate the role of PARK7 in the oxidative stress-induced mucosal permeability. Oxidative stress induced by H_2_O_2_ increased the permeability of the sacs derived from the control or Comp23-pretreated mice. However, Comp23 pretreatment decreased the permeability of sacs whether treated with H_2_O_2_ or not compared to the corresponding sacs derived from the untreated control mice (Figure 6).

## 4. Discussion

Oxidative stress and consequent accumulation of damaged intracellular macromolecules [19, 20] are important components of mucosal deterioration and increased intestinal permeability of patients with CD [21]. Recently, Moretti et al. reported that the production of ROS strictly correlates with the severity of intestinal damage; therefore, they recommended the routine determination of serum antioxidant capacity to monitor the efficiency of the gluten-free diet of patients with CD [22].

Previously, our research group demonstrated the increased presence of PARK7 in the small intestinal mucosa of children with CD [8]. PARK7 is an ubiquitously expressed antioxidant protein, in which significance is clearly demonstrated in the pathomechanism of different neurodegenerative diseases [12]. However, our knowledge is limited about the possible role of PARK7 in the preservation of the epithelial layer integrity in the inflamed intestine.

In the present study, in accordance with our previous study [8], we found that PARK7 is abundantly present in the duodenal epithelial cells of therapy-naive children with CD, and also in FHs74Int duodenal epithelial cells (Figure 1).

Therefore, in the following experiments, FHs74Int cells exposed to oxidative stress were used to investigate the role of PARK7 in the maintenance of mucosal integrity. In these experiments, Comp23, a PARK7-binding compound developed by Kitamura et al. [23, 24], was used to enhance the antioxidant activity of PARK7. They suggest that Comp23 can prevent the excessive oxidation of PARK7 by binding to its C106 cysteine residue, thereby preserving the antioxidant function of PARK7.

In the first set of experiments, we found that Comp23 decreased the intracellular accumulation of ROS in H_2_O_2_-treated FH74Int cells (Figure 2). Our result is in line with previous studies demonstrating that treatment with recombinant PARK7 decreased the oxidative stress-induced ROS accumulation in neuronal cells [25, 26]. Similarly, previous studies also demonstrated that the lack of PARK7 is associated with enhanced ROS accumulation in various cell types proposing the determinative role of PARK7 in the antioxidant defence [27–29].

Investigating the molecular mechanism by which PARK7 may modulate the oxidative stress, we found that Comp23 treatment increased the expression of nuclear factor erythroid 2-related factor 2 (*NRF2*) transcription factor, which is known as one of the major regulators of cellular antioxidant defence. In accordance, we found that Comp23 treatment increased the expression of a multitude of NRF2-dependent antioxidant response genes, including thioredoxin (*TRX1*), *γ*-glutamyl cysteine synthetase (*GCLC*), heme oxygenase (*HMOX1*), and NAD(P)H:quinone oxidoreductase 1 (*NQO1*) of the H_2_O_2_-treated FHs74Int cells (Figure 2). Our findings, in line with the literature, strongly suggest that Comp23 significantly influences the activation of the PARK7-NRF2 axis thereby influencing the consequence of oxidative stress [30, 31]. Indeed, previously, it has been shown that the activation of the NRF2 pathway attenuates the stress-related apoptosis in numerous cell types [32–34].

In accordance with decreased intracellular ROS accumulation and increased antioxidant preparedness of the epithelial cells, we demonstrated that Comp23 treatment improved the survival of H_2_O_2_-treated cells (Figure 3). Our finding is in accordance with the results of Kitamura et al., describing for the first time the PARK7-dependent protective effect of Comp23 against H_2_O_2_-induced cell death in neuroblasts [23, 24]. Investigating the possible molecular mechanism, we found that Comp23 treatment increased the expression of tumor antigen P53 (*TP53*) transcription factor and its target genes [35], including cyclin-dependent kinase inhibitor 1-p21 (*CDKN1A*), proliferating cell nuclear antigen (*PCNA*), apoptosis regulator Bcl-2 (*BCL2*), and apoptosis regulator BAX (*BAX*) (Figure 3). These results are in accordance with the previous findings, demonstrating that PARK7 modulates P53-P21 pathway in an oxidation status-dependent manner [36]. P53 is a determinative regulator of the cell cycle, having a dual function during stress response as it prevents intracellular oxidation to ensure cell survival and repair at a low level of damage, but it also can induce apoptosis in case of severe cellular injury [37, 38]. Taken together, our results suggest that the antiapoptotic effect of Comp23 is owing to the combined activation of NRF2 and P53 pathways.

In the next set of experiments, we investigated the role of PARK7 in the maintenance of mucosal integrity of CD patients. Investigating the duodenal biopsies of CD patients, we found that the actin cytoskeleton of epithelial cells is seriously injured. Indeed, we found that the well-contoured structure of the actin belts, a characteristic for the healthy cells, collapsed resulting in granular dots in the epithelial cells of inflamed mucosa (Figure 4).

Examining the effect of oxidative stress on FHs74Int cells, we found that similarly to the duodenal epithelial cells of the inflamed mucosa, the H_2_O_2_ treatment destroyed the filamentous actin network of the cells, resulting in dot-like aggregates (Figure 4). The oxidative damage also decreased the expression of CAMs, including zonula occludens 1 (*ZO1*), *CDH1*, *VCL*, and *ITGB5*, and led to the dissociation of ZO-1 from the cytoskeleton of the cells (Figure 5). Our observation is in line with previous literary knowledge about the oxidative stress-induced structural and functional modification of intracellular macromolecules, including filamentous actin resulting in its depolymerisation and thereby the disruption of the physiological cytoskeleton [39]. Actin filaments are associated with the intracellular localisation of CAMs, including cadherins, tight-junction, and focal adhesion proteins [40, 41], ensuring the stable cell surface and cell-cell adhesion and thereby the maintenance of the mucosal barrier function [42, 43]. Interestingly, we also observed that the Comp23 treatment increased the expression of CAMs and, more importantly, preserved the healthy architecture of the actin cytoskeleton-CAM complexes in the epithelial cells exposed to oxidative stress (Figures 4 and 5).

Therefore, to further investigate the significance of PARK7 in the maintenance of the mucosal integrity, we examined its effect on the permeability of H_2_O_2_-treated small intestinal tissue preparations *ex vivo*. According to our previous *in vitro* experiments, we found that Comp23 greatly decreased the permeability of the small intestinal sacs (Figure 6), confirming that PARK7 has a determinative role in the control of oxidative stress and in the maintenance of mucosal integrity. The damage of the intestinal barrier leads to increased penetration of luminal noxious molecules, including gliadin or luminal bacteria into the lamina propria of the intestine thereby exacerbating mucosal inflammation of patients with CD [40]. Therefore, the preservation of the intestinal barrier may offer a new therapeutic approach to inhibit intestinal inflammation in CD patients.

## 5. Conclusion

**Figure 7:**
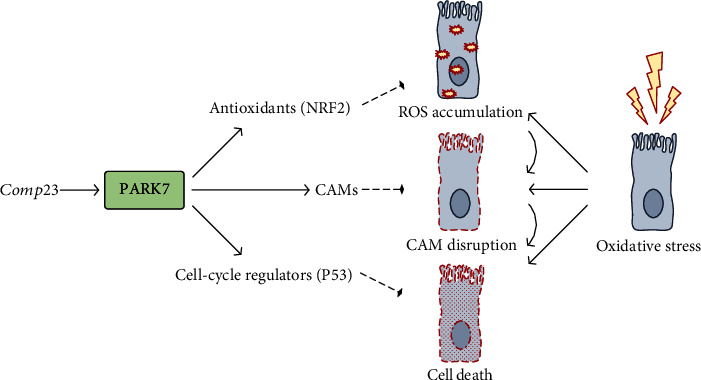
Schematic model of the proposed mechanism underlying the protective effect of PARK7 against oxidative damage of duodenal epithelial cells in celiac disease. Our results showed that treatment with Comp23, a PARK7-binding compound under oxidative stress conditions, results in enhanced transcriptional activity, inducing expression of stress-response elements, such as antioxidant or cell-cycle regulators. This effect of PARK7 leads to reduced cellular damage and improved cell adhesion, contributing to the maintenance of integrity in the inflamed mucosa. Comp23: PARK7-binding compound; ROS: reactive oxygen species; CAM: cell adhesion molecule.

In summary, we demonstrated the protective effect of the PARK7-binding Comp23 in the duodenal epithelial cells exposed to oxidative stress. We showed that PARK7 induced the expression of stress-response elements, including antioxidant and cell-cycle regulator genes, and normalized the expression of CAMs in the epithelial cells exposed to oxidative stress (Figure 7). These processes contribute to the maintenance of mucosal integrity as demonstrated by our *ex vivo* experiment and thereby may moderate the small intestinal inflammation in CD.

Although there are limitations of our study, including the lack of quantitative measurement at the protein level, we hope that our study made a great progress in the understanding of the biological effects of PARK7 in the pathomechanism of CD. Moreover, our present work may contribute not only to the better understanding of the pathomechanism of CD, but also to the development of new therapeutic approaches.

## Figures and Tables

**Table 1:** Nucleotide sequences of primer pairs applied for the real-time polymerase chain reaction (RT-PCR) detection. Abbreviations: ref. seq.: reference sequence; F: forward; R: reverse.

Gene	NCBI ref. seq.	Primer pairs	
*BAX*	NM_001291428.2	F:	5′- GGA TGA TTG CCG CCG TGG ACA CAG -3′
		R:	5′-CAA CAG CCG CTC CCG GAG GAA GTC-3′

*BCL2*	NM_000633.2	F:	5′-CGG GGT GAA CTG GGG GAG GAT TGT-3′
		R:	5′-AGG TGT GCA GGT GCC GGT TCA GGT-3′

*CDH1*	NM_004360.4	F:	5′-AAGGAGGCGGAGAAGAGGACCAG-3′
		R:	5′-GAT TGG CAG GGC GGG GAA G-3′

*CDKN1A*	NM_001220777.1	F:	5′-TTG TAC CCT TGT GCC TCG CTC AGG-3′
		R:	5′-ATC AGC CGG CGT TTG GAG TGG TAG-3′

*GCLC*	NM_001498.4	F:	5′-AAA AGT CCG GTT GGT CCT GTC TGG-3′
		R:	5′-GGC TGT CCT GGT GTC CCT TCA ATC-3′

*HMOX1*	NM_002133.2	F:	5′-CCA GCG GGC CAG CAA CAA AG-3′
		R:	5′-TGT CGC CAC CAG AAA GCT GAG TGT-3′

*ITGB5*	NM_002213.5	F:	5′- TCC GCC ATC TGC TGC CTC TCA C-3′
		R:	5′-CAT CCT TTC GCC AGC CAA TCT TCT C-3′

*NQO1*	NM_000903.3	F:	5′-CTG CTG CAG CGG CTT TGA AGA-3′
		R:	5′-GCC AGA ACA GAC TCG GCA GGA TAC-3′

*NRF2*	NM_006164.4	F:	5′-CAG CAG GAC ATG GAT TTG ATT G-3′
		R:	5′-ACT GGT TTC TGA CTG GAT GTG CT-3′

*PCNA*	NM_002592.2	F:	5′-GCG GTC TGA GGG CTT CGA CAC CTA-3′
		R:	5′-CCG CGT TAT CTT CGG CCC TTA GTG-3′

*RN18S*	HQ387008.1	F:	5′-GGC GGC GAC GAC CCA TTC-3′
		R:	5′-TGG ATG TGG TAG CCG TTT CTC AGG-3′

*TP53*	NM_001126118.1	F:	5′-TGG TCT GGC CCC TCC TCA GCA TCT-3′
		R:	5′-TCA GGC GGC TCA TAG GGC ACC AC-3′

*TRX1*	NM_003329.3	F:	5′-ATG CAT GCC AAC ATT CCA GTT TT-3′
		R:	5′-ATG GTG GCT TCA AGC TTT TCC TTA-3′

*VCL*	NM_014000.2	F:	5′-CCA CGG CGC CTC CTG ATG C-3′
		R:	5′-GGC CTG AAT GCC TTC CAC TGT TGA-3′

*ZO1*	NM_021258.3	F:	5′-ACC ACA AGC GCA GCC ACA ACC AAT-3′
		R:	5′-GGG GTG GGC TCC TCC AGT CTG ACA T-3′

## Data Availability

The data used to support the findings of this study are included in the article.

## Abbreviations

CAM: Cell adhesion molecule
CD: Celiac disease
DCFDA: Dichlorofluorescein diacetate
FBS: Fetal bovine serum
LDH: Lactate dehydrogenase
PARK7: Parkinson's disease 7
PBS: Phosphate-buffered saline
ROS: Reactive oxygen species
RT: Room temperature.

## References

[1] Ludvigsson J. F., Leffler D. A., Bai J. C. (2012). The Oslo definitions for coeliac disease and related terms. *Gut*.

[2] Ferretti G., Bacchetti T., Masciangelo S., Saturni L. (2012). Celiac disease, inflammation and oxidative damage: a nutrigenetic approach. *Nutrients*.

[3] Ramachandran A., Madesh M., Balasubramanian K. A. (2001). Apoptosis in the intestinal epithelium: its relevance in normal and pathophysiological conditions. *Journal of Gastroenterology and Hepatology*.

[4] Günther C., Neumann H., Neurath M. F., Becker C. (2013). Apoptosis, necrosis and necroptosis: cell death regulation in the intestinal epithelium. *Gut*.

[5] Bonifati V., Rizzu P., van Baren M. J. (2002). Mutations in the DJ-1 gene associated with autosomal recessive early-onset parkinsonism. *Science*.

[6] Kahle P. J., Waak J., Gasser T. (2009). DJ-1 and prevention of oxidative stress in Parkinson’s disease and other age-related disorders. *Free Radical Biology and Medicine*.

[7] Ariga H., Iguchi-Ariga S. M. M. (2017). *DJ-1/PARK7 Protein: Parkinson’s Disease, Cancer and Oxidative Stress-Induced Diseases*.

[8] Vörös P., Sziksz E., Himer L. (2013). Expression of PARK7 is increased in celiac disease. *Virchows Archiv*.

[9] Dubois P. C. A., Trynka G., Franke L. (2010). Multiple common variants for celiac disease influencing immune gene expression. *Nature Genetics*.

[10] Anderson C. A., Boucher G., Lees C. W. (2011). Meta-analysis identifies 29 additional ulcerative colitis risk loci, increasing the number of confirmed associations to 47. *Nature Genetics*.

[11] Saito Y. (2014). Oxidized DJ-1 as a possible biomarker of Parkinson’s disease. *Journal of Clinical Biochemistry and Nutrition*.

[12] Hijioka M., Inden M., Yanagisawa D., Kitamura Y. (2017). DJ-1/PARK7: a new therapeutic target for neurodegenerative disorders. *Biological and Pharmaceutical Bulletin*.

[13] Fasano A., Araya M., Bhatnagar S. (2008). Federation of International Societies of Pediatric Gastroenterology, Hepatology, and Nutrition consensus report on celiac disease. *Journal of Pediatric Gastroenterology and Nutrition*.

[14] Mateer S. W., Cardona J., Marks E., Goggin B. J., Hua S., Keely S. (2016). *Ex vivo* intestinal sacs to assess mucosal permeability in models of gastrointestinal disease. *JoVE (Journal of Visualized Experiments)*.

[15] Lange S., Delbro D. S., Jennische E. (2009). Evans blue permeation of intestinal mucosa in the rat. *Scandinavian Journal of Gastroenterology*.

[16] Korzeniewski C., Callewaert D. M. (1983). An enzyme-release assay for natural cytotoxicity. *Journal of Immunological Methods*.

[17] Gonzalez A., Santofimia-Castaño P., Rivera-Barreno R., Salido G. M. (2012). Cinnamtannin B-1, a natural antioxidant that reduces the effects of H_2_O_2_ on CCK-8-evoked responses in mouse pancreatic acinar cells. *Journal of Physiology and Biochemistry*.

[18] Livak K. J., Schmittgen T. D. (2001). Analysis of relative gene expression data using real-time quantitative PCR and the 2− *ΔΔ*CT method. *Methods*.

[19] Odetti P., Valentini S., Aragno I. (2009). Oxidative stress in subjects affected by celiac disease. *Free Radical Research*.

[20] Szaflarska-Popławska A., Siomek A., Czerwionka-Szaflarska M. (2010). Oxidatively damaged DNA/oxidative stress in children with celiac disease. *Cancer Epidemiology and Prevention Biomarkers*.

[21] Diosdado B., van Oort E., Wijmenga C. (2005). “Coelionomics”: towards understanding the molecular pathology of coeliac disease. *Clinical Chemistry and Laboratory Medicine*.

[22] Moretti S., Mrakic-Sposta S., Roncoroni L. (2018). Oxidative stress as a biomarker for monitoring treated celiac disease. *Clinical and Translational Gastroenterology*.

[23] Kitamura Y., Watanabe S., Taguchi M. (2011). Neuroprotective effect of a new DJ-1-binding compound against neurodegeneration in Parkinson’s disease and stroke model rats. *Molecular Neurodegeneration*.

[24] Takahashi-Niki K., Inafune A., Michitani N. (2015). DJ-1-dependent protective activity of DJ-1-binding compound no. 23 against neuronal cell death in MPTP-treated mouse model of Parkinson’s disease. *Journal of Pharmacological Sciences*.

[25] Yanagisawa D., Kitamura Y., Inden M. (2007). DJ-1 protects against neurodegeneration caused by focal cerebral ischemia and reperfusion in rats. *Journal of Cerebral Blood Flow & Metabolism*.

[26] Inden M., Taira T., Kitamura Y. (2006). PARK7 DJ-1 protects against degeneration of nigral dopaminergic neurons in Parkinson’s disease rat model. *Neurobiology of Disease*.

[27] Singh Y., Chen H., Zhou Y. (2016). Differential effect of DJ-1/PARK7 on development of natural and induced regulatory T cells. *Scientific Reports*.

[28] Yang J., Kim M. J., Yoon W. (2017). Isocitrate protects DJ-1 null dopaminergic cells from oxidative stress through NADP+-dependent isocitrate dehydrogenase (IDH). *PLoS Genetics*.

[29] Shi S. Y., Lu S.-Y., Sivasubramaniyam T. (2015). DJ-1 links muscle ROS production with metabolic reprogramming and systemic energy homeostasis in mice. *Nature Communications*.

[30] Ma Q. (2013). Role of nrf2 in oxidative stress and toxicity. *Annual Review of Pharmacology and Toxicology*.

[31] Raninga P. V., Di Trapani G., Tonissen K. F. (2017). The multifaceted roles of DJ-1 as an antioxidant. *DJ-1/PARK7 Protein*.

[32] Chung S. D., Lai T. Y., Chien C. T., Yu H. J. (2012). Activating Nrf-2 signaling depresses unilateral ureteral obstruction-evoked mitochondrial stress-related autophagy, apoptosis and pyroptosis in kidney. *PLoS One*.

[33] Mohammadzadeh M., Halabian R., Gharehbaghian A. (2012). Nrf-2 overexpression in mesenchymal stem cells reduces oxidative stress-induced apoptosis and cytotoxicity. *Cell Stress and Chaperones*.

[34] Khan N. M., Ahmad I., Haqqi T. M. (2018). Nrf2/ARE pathway attenuates oxidative and apoptotic response in human osteoarthritis chondrocytes by activating ERK1/2/ELK1-P70S6K-P90RSK signaling axis. *Free Radical Biology & Medicine*.

[35] Wei C.-L., Wu Q., Vega V. B. (2006). A global map of p53 transcription-factor binding sites in the human genome. *Cell*.

[36] Kato I., Maita H., Takahashi-Niki K. (2012). Oxidized DJ-1 inhibits p53 by sequestering p53 from promoters in a DNA-binding affinity-dependent manner. *Molecular and Cellular Biology*.

[37] Bensaad K., Vousden K. H. (2007). p53: new roles in metabolism. *Trends in Cell Biology*.

[38] Liu D., Xu Y. (2011). p53, oxidative stress, and aging. *Antioxidants & Redox Signaling*.

[39] Rao R. (2008). Oxidative stress-induced disruption of epithelial and endothelial tight junctions. *Frontiers in Bioscience*.

[40] Ivanov A. I., Parkos C. A., Nusrat A. (2010). Cytoskeletal regulation of epithelial barrier function during inflammation. *The American Journal of Pathology*.

[41] Wang D., Naydenov N. G., Feygin A., Baranwal S., Kuemmerle J. F., Ivanov A. I. (2016). Actin-depolymerizing factor and cofilin-1 have unique and overlapping functions in regulating intestinal epithelial junctions and mucosal inflammation. *The American Journal of Pathology*.

[42] Lee S. H. (2015). Intestinal permeability regulation by tight junction: implication on inflammatory bowel diseases. *Intestinal Research*.

[43] Higashiyama T., Katsuyama A., Otori H. (2014). Detection of cellular damage by hydrogen peroxide using SV40-T2 cells on shear horizontal surface acoustic wave (SH-SAW) sensor. *Ultrasonics*.

